# The more the merrier: Conspecific density improves performance of gregarious larvae and reduces susceptibility to a pupal parasitoid

**DOI:** 10.1002/ece3.3571

**Published:** 2017-11-07

**Authors:** Elena Rosa, Saskya van Nouhuys, Marjo Saastamoinen

**Affiliations:** ^1^ Department of Biosciences Metapopulation Research Centre University of Helsinki Helsinki Finland; ^2^ Department of Entomology Cornell University Ithaca NY USA

**Keywords:** aggregation, density, genetic variation, immunity, Lepidoptera, *Melitaea cinxia*, parasitoid, *Pteromalus apum*

## Abstract

Aggregation can confer advantages in animal foraging, defense, and thermoregulation. There is a tight connection between the evolution of insect sociality and a highly effective immune system, presumably to inhibit rapid disease spread in a crowded environment. This connection is less evident for animals that spend only part of their life cycle in a social environment, such as noneusocial gregarious insects. Our aim was to elucidate the effects of group living by the gregarious larvae of the Glanville fritillary butterfly with respect to individual performance, immunity, and susceptibility to a parasitoid. We were also interested in the role of family relative to common postdiapause environment in shaping life‐history traits. Larvae were reared at high or low density and then exposed to the pupal parasitoid wasp *Pteromalus apum*, either in presence or absence of a previous immune challenge that was used to measure the encapsulation immune response. Surviving adult butterflies were further tested for immunity. The wasp offspring from successfully parasitized butterfly pupae were counted and their brood sex ratios assessed. Larvae reared at high density grew larger and faster than those at low density. Despite high mortality due to parasitism, survival was greater among individuals with high pupal immunity in both density treatments. Moreover, butterfly pupae reared at high density were able to kill a larger fraction of individuals in the parasitoid broods, although this did not increase survival of the host. Finally, a larger proportion of variation observed in most of the traits was explained by butterfly family than by common postdiapause rearing environment, except for adult survival and immunity, for which this pattern was reversed. This gregarious butterfly clearly benefits from high conspecific density in terms of developmental performance and its ability to fight a parasitoid. These positive effects may be driven by cooperative interactions during feeding.

## 1 INTRODUCTION

Many animals form conspecific aggregation. Several potential advantages of this behavior have been found, ranging from predator avoidance to foraging and dispersal efficiency, thermoregulation, or a combination of these (see Krause & Ruxton, 2002 for details). However, aggregation can also be costly, as a group can easily be detected by predators and infections spread rapidly in a crowded environment (Arneberg, Skorping, Grenfell, & Read, 1998). In addition, resource competition among group members may occur (Parrish & Edelstein‐Keshet, 1999). For many species, group aggregation occurs only during particular activities or life cycle stages. For instance, some animals forage in herds, and birds migrate in flocks (Parrish & Edelstein‐Keshet, 1999).

**Figure 1:**
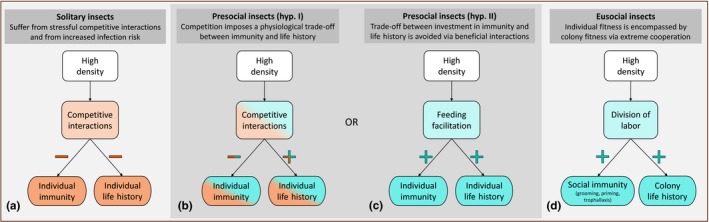
The relationship between immunity and life‐history traits when group living is experienced by insects with different life histories (with ad libitum food): solitary (a), presocial (b and c), and eusocial (d). Positive and negative interactions are shown in blue and orange, respectively, and trade‐offs are indicated by shading of the two colors. Solitary insects are expected to compete, resulting in reduced investment in both immunity and life‐history traits when experimentally reared under crowded conditions (a), as suggested by previous work (Piesk et al., 2013; Reilly & Hajek, 2008; Wilson et al., 2003). Eusocial insects are expected to benefit in terms of both immunity and life history under high conspecific density (d). Two hypotheses of how conspecific density may be experienced by presocial insects are depicted: (b) Competition among conspecifics triggers general stress responses, which can promote investment in immunity or in life‐history traits, but with a trade‐off between them; (c) cooperation among conspecifics (e.g., feeding facilitation) is beneficial for both life history and investment in immunity, with no trade‐off

Like vertebrates, insects include species that are completely solitary and species with extremely organized societies. The benefits of both these extremes are intuitive, as solitary insects appear to not engage in crowding and thus completely avoid its costs, whereas eusocial insects make the most out of crowding by increasing synergy. Eusocial insects have evolved sophisticated mechanisms to exploit the potential of crowding via extreme organization including division of labor and social immunity (Hughes, Eilenberg, & Boomsma, 2002; Traniello, Rosengaus, & Savoie, 2002) (Figure 1d). In these societies, the benefits of crowding outweigh the costs of increased infection risk and competition. In fact, mechanisms evolved by social insects, such as immune priming, grooming, and trophallaxis (Rosengaus, Malak, & MacKintosh, 2013), preventively account for the increased infection risk promoted by crowding. This makes the upregulation of social immunity one of the reasons for the success of eusociality (Cremer, Armitage, & Schmid‐Hempel, 2007). Conversely, solitary insects minimize the main costs of crowding by avoiding aggregation entirely (Figure 1a). Costs of crowding can be direct, such as increased risk of infection, and competition increasing physical injury and cannibalism (Mason, Cannizzo, & Raffa, 2014; Srygley, 2012). In addition, physiological trade‐offs between investment in expensive immune responses and other life‐history traits are also crucial (Sheldon & Verhulst, 1996; Zuk & Stoehr, 2002), as suggested by solitary Lepidoptera having low immunity or high mortality under experimental crowding (Piesk, Karl, Franke, & Fischer, 2013; Reilly & Hajek, 2008), or by the lack of evidence of inherently solitary species showing improved immunity under crowded conditions (Wilson, Knell, Boots, & Koch‐Osborne, 2003).

Between the extreme cases of eusocial and solitary, we find many species that aggregate occasionally and exhibit intermediate life histories. However, the ecological mechanisms underlying these intermediate conditions, and the respective life‐history responses to them, are not well understood. Insects living together during a specific stage of their life cycle, such as some bee, wasp, beetle species, and gregarious lepidopteran larvae, are traditionally classified as “presocial” (Costa, 1997; Fitzgerald & Costa, 1999). Other insects aggregate occasionally only if they experience certain conditions, such as crowding during juvenile stages, and are known as “phase‐polyphenic” species (Applebaum & Heifetz, 1999). Some phase‐polyphenic insects have evolved a plastic response to anticipate the increased infection risk due to crowding, which consists of upregulating their immunity when in the gregarious phase (i.e., density‐dependent prophylaxis, Wilson & Reeson, 1998; Reeson, Wilson, Gunn, Hails, & Goulson, 1998; reviewed in Wilson & Cotter, 2009). These examples suggest that the infection risk caused by recurrent crowding has presumably led to the evolution of a plastic immune system, similar to that of eusocial insects, but less structured. These findings suggest that the regulation of the immune system of species with intermediate life histories should share some characteristics with both social and solitary species.

We propose to test two alternative hypotheses to explain how a presocial insect species may represent an intermediate evolutionary state between solitary and an entirely social life history. Hypothesis (I): Crowding causes competitive interactions even under ad libitum food conditions. Under this condition, we expect a physiological trade‐off (Sheldon & Verhulst, 1996) between investment in immunity and life‐history traits (Figure 1b). Physical interactions in crowded conditions may upregulate the baseline general stress responses and make the immune system more reactive, but consequently reduce resource allocation to life‐history traits. Alternatively, the stressful interactions may push the conspecifics to consume more food and grow more, leading to reduced investment in immune defenses. We suggest that under this condition, group living is “tolerated” due to the potential benefits, such as thermoregulation and collective degradation of leaf material. Hypothesis (II): Crowding is beneficial for conspecifics because it reduces the physiological cost of thermoregulating and favors cooperative interactions such as feeding facilitation (Fitzgerald & Costa, 1999). Due to these benefits, there is an increase in the resources available for investment among life‐history traits, and stressful interactions are avoided. Hence, under this condition, we expect no trade‐off between immunity and life‐history traits (Figure 1c).

Another important feature of many gregarious and social insects is the common environment shared among nest mates. While the genetic background is usually the same among members of a gregarious group, individuals from the same family do not always share the same environment. For example, when full‐sibling broods are oviposited at different sites, the life history of each brood may be critically shaped by the local environment irrespective of the common genetic background. The local environment can have important implications for insect immunity, as some species spread antimicrobial compounds inside their colony, or through trophallaxis and cooperative hygienic behaviors (Hamilton, Lejeune, & Rosengaus, 2011; Rosengaus, Maxmen, Coates, & Traniello, 1998; Simone, Evans, & Spivak, 2009).

We used the presocial Glanville fritillary butterfly, *Melitaea cinxia* (Lepidoptera: Nymphalidae) as model system to assess responses to crowding, and more specifically to test how postdiapause larval density impacts life‐history trade‐offs between development and immunity (hypothesis I vs. II, Figure 1). Glanville fritillary larvae are gregarious and develop in mainly full‐sibling groups of variable size. In order to address the effects of crowding on postdiapause larvae, we manipulated the degree of conspecific density by imposing a “high‐” and a “low”‐density treatment under laboratory conditions. Once they pupated, we exposed them to the parasitoid wasp *Pteromalus apum* (Hymenoptera: Pteromalidae) with or without a previous immune challenge through wounding with a nylon filament. Degree of encapsulation of the filament was then used as an assay of insect immune response. Our four main objectives were to determine the following: (i) the effect of density after diapause on the performance of larvae and pupae; (ii) the impact of larval density on immune defense and susceptibility to parasitism, and the presence of immune priming in *M. cinxia*; (iii) whether rearing density, or the putatively enhanced encapsulation ability via immune priming, could impair development of the parasitoid brood; and (iv) the amount of phenotypic variation explained by the larval family (genetic background), compared to the variation explained by common postdiapause environment of larval groups, or by parasitoid genetic background.

## 2 MATERIALS AND METHODS

### 2.1 Study species

In Finland, the Glanville fritillary butterfly occurs solely in the Åland archipelago, where it persists as a classic metapopulation (Hanski, 1998). The habitat of the butterfly is dry meadows or pastures with one or both larval host plants, *Plantago lanceolata* and *Veronica spicata*. The adults fly from early–June until early–July and females lay clusters of 50–250 eggs on a host plant, from which the larvae hatch in 2–3 weeks (Ojanen, Nieminen, Meyke, Pöyry, & Hanski, 2013). Prediapause larvae spend up to five instars feeding gregariously on the host plant, and in the fall, they spin a silken winter nest, where they diapause gregariously until March or April. Group size can vary depending on maternal condition, larval mortality due to starvation, predation and parasitism, and to weather during overwintering. Under field conditions, survival of eggs and larvae before and during diapause has been shown to increase with group size (Kuussaari & Singer, 2017). After snowmelt, larvae terminate their diapause and start feeding gregariously, although groups may split or merge depending on the resources available. During the 7th and usually final instar, larvae start moving in search of host plants and may split into small groups or become solitary, until they pupate in May (Ojanen et al., 2013). Adult eclosion occurs after 2–4 weeks.

**Figure 2:**
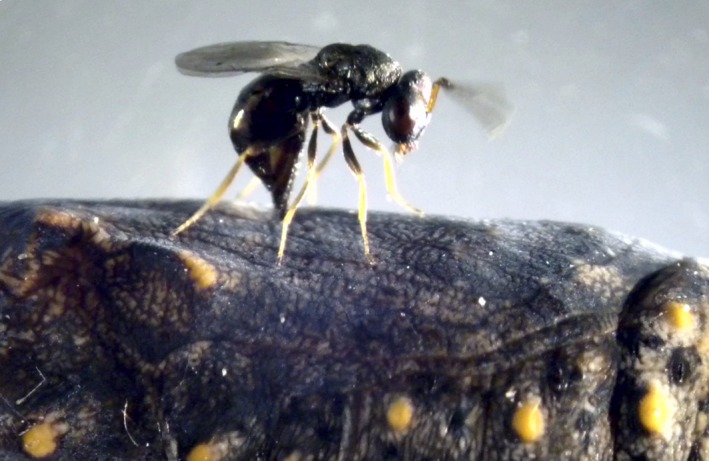
A female of *Pteromalus apum* ovipositing into the puparial wall of a Glanville fritillary. Photograph by S. van Nouhuys

In Finland, the Glanville fritillary is the host of several parasitoids, two of which, *Cotesia melitaearum* (Braconidae: Microgastrinae) and *Hyposoter horticola* (Ichneumonidae: Campopleginae), are specialists of *M. cinxia* larvae. At least four parasitoid species are known to attack the pupae, of which the most common is *P. apum* (Chalcidoidea: Pteromalidae; Figure 2), which parasitizes up to half *M. cinxia* pupae (Lei, Vikberg, Nieminen, & Kuussaari, 1997; van Nouhuys & Hanski, 2002). This generalist parasitoid wasp is mostly associated with Nymphalid butterfly pupae (Shaw, Stefanescu, & van Nouhuys, 2009). In the Åland Islands, *P. apum* parasitizes *M. cinxia* and the closely related *Melitaea athalia* (Kraft & van Nouhuys, 2013). *Pteromalus apum* is an ecto‐parasitoid laying eggs in the interior part of the puparial wall; hence, eggs are not directly in contact with the host hemocoel. Eggs develop inside the host pupal case and juveniles feed on the pupal hemolymph. When they become adults, they exit from a hole in the pupal case. Brood size ranges between 1 and 60 per host (Kraft & van Nouhuys, 2013) with larger mixed broods due to superparasitism (van Nouhuys & Kraft, 2012). Individuals are 1–2 mm long and mate immediately; hence, sib mating is frequently observed under laboratory conditions (Kraft & van Nouhuys, 2013).

### 2.2 Experimental setup

To obtain a laboratory generation of pupal parasitoids, 78 potted host plants (*P. lanceolata*) were taken to eight meadows inhabited by *M. cinxia* in Åland. The meadows were chosen based on parasitoid abundances recorded in previous years (SvN, personal observation). Each potted plant hosted two last instar *M. cinxia* larvae from a laboratory maintained colony. The plants were covered with a coarse mesh to prevent larvae from escaping, yet allow the tiny *P. apum* females to enter and parasitize the hosts once they had pupated. The host plants were checked periodically and collected after 18–25 days, depending on when the *M. cinxia* pupated. The pupae were put individually in petri dishes and after ~15 days in the laboratory wasps emerged. About 10% of the pupae put in the field were parasitized.

The *M. cinxia* larvae used for the experiment were from 11 full‐sibling families from a laboratory colony. After hatching, each family was reared in constant laboratory conditions (26°C day: 10°C night) until diapause. They spent the whole diapause in the dark at ~5°C until the following spring.

#### 2.2.1 Effect of high rearing density on larval performance (i)

Once woken up from the diapause, larvae from each family were randomly divided into two density treatments: high density (HD) and low density (LD). High‐density treatment consisted of 12 larvae per container (10 × 13 × 4 cm), whereas LD treatment consisted of four larvae per container. To have comparable sample sizes in both density treatments, there were three times as many replicates per family in the low density as there were for the high density. The containers were kept in controlled light and temperature conditions in a SANYO climate chamber (27°C day: 10°C night, 12:12 hr). Larvae were fed daily on a leaf mixture of the two host plants (*P. lanceolata* and *V. spicata*), ensuring ad libitum availability of food in both treatment groups. While providing food, the general conditions of the larvae were checked and dead individuals removed. Daily operations were performed under sterile conditions to avoid stress to the larvae and minimize disease spread. Larval mortality during the experiment was quite high (45%), possibly due to stress related to being slightly longer in diapause than usual (i.e., ~8.5 months; the usual diapause under laboratory conditions is 6–8 months). The duration of diapause was dictated by the necessity of synchronizing butterfly pupation with the emergence of pupal parasitoids in the wild, which in turn was delayed by abnormally rainy weather in spring 2015.

#### 2.2.2 Effect of high larval density on butterfly immunity, susceptibility to a parasitoid, and potential immune priming (ii)

Upon pupation, individuals were weighed and further divided into four treatments: naïve (*N*), encapsulation only (*E*), parasitism only (*P*), and encapsulation followed by parasitism (*EP*). The latter treatment group was designed to assess potential immune priming. Immune priming has been documented in invertebrates, and in insects it can take place against dead bacteria, components of bacterial cellular wall, or sick nest mates (reviewed in Little & Kraaijeveld, 2004; Best, Tidbury, White, & Boots, 2013). Similarly, it may facilitate the future defense against parasitoids through a prior wounding challenge mimicking a parasitoid attack (Erler, Popp, & Lattorff, 2011; Han, Chun, Schwartz, Nelson, & Paskewitz, 1999; Krams et al., 2016). The typical immune reaction activated by wounding or parasitism is the encapsulation response (e.g., Laurentz et al., 2012) in which plasmatocytes and granular cells are layered around macro parasites and some micro pathogens in the insect hemolymph (Gillespie, Kanost, & Trenczek, 1997; Pech & Strand, 1996). The final step of this process consists of the melanization of the capsule (Marmaras, Charalambidis, & Zervas, 1996). Naïve pupae were left untreated until eclosion, whereas pupae of *E* and *EP* groups underwent the encapsulation immune assay during the morning of the third day after pupation. Pupae of groups *P* and *EP* were parasitized by *P. apum* in the afternoon of the third day after pupation (i.e., 4 hr after the encapsulation assay). All pupae were then left untouched until eclosion of the adult butterfly or the parasitoid wasps at room temperature (25 ± 3°C day: 21 ± 3°C night). Surviving 1‐day‐old butterflies underwent the encapsulation immune assay again.

#### 2.2.3 Effect of high density and potential priming on parasitoid brood performance (iii)

It is known from field experiments at a larger spatial scale that *M. cinxia* density influences parasitism rate and brood sex ratio of *P. apum* (Kraft & van Nouhuys, 2013). To assess the brood size and sex ratio of parasitoid wasps, eclosing offspring from successfully parasitized pupae were counted and sexed. All noneclosed butterfly pupae (from which parasitoids eclosed and from which neither parasitoids nor butterflies eclosed) were dissected to estimate the number of dead parasitoids within each pupa.

#### 2.2.4 Amount of phenotypic variation explained by larval family (iv)

The 11 larval families used for the experiments were equally divided among the two density treatments during the postdiapause stage and, depending on the size of the family, we made at least two replicates (i.e., larval containers) of each treatment within a family. Individuals within a container were equally divided among the four pupal treatments. This allowed us to estimate statistically the amount of variance explained by larval container and family for objectives (i) and (ii). The location of origin of parasitoid wasps was also known. The proportion of variance explained by wasp origin with respect to larval family was estimated for objective (iii).

### 2.3 Immunity measures

Pupal and adult encapsulation assays were performed following methods by Saastamoinen and Rantala (2013), in which a 2‐mm‐long nylon filament is inserted in the pupal or adult cuticle for 1 hr. After removal, the filament was frozen at −20°C and then photographed from three different angles in constant light conditions. Digital images were analyzed with the software ImageJ (version 1.47), and melanization was measured as mean gray values of reflecting light per filament.

### 2.4 Parasitism

*Pteromalus apum* females from broods reared from the *M. cinxia* pupae that had been placed in the field were used for parasitism (*P* and *EP* treatments). Sib mating was observed for all the wasp broods immediately after eclosion, ensuring that the females used in the parasitism assay were mated. Parasitism was performed at the same time in the afternoon for all the individuals, on the third day after pupation. For the parasitism, a single female wasp was put in a petri dish with one host pupa. The petri dishes were observed for 2 hr, and *P. apum* females ovipositing into the pupae were scored. Wasps that were inactive during the first hour were replaced by a new wasp. To ensure parasitism, parasitoids and pupae were left in the petri dish for 24 hr, after which parasitoids were removed. Individual wasps were maintained by being fed a 1:2 honey water solution and reused for parasitism a maximum of three times with intervals of at least 48 hr between subsequent parasitism events. The average development time to reach adulthood was 7–8 days for butterflies, and 14–15 days for parasitoid broods. Butterfly pupae from which no butterfly or parasitoids eclosed after 20 days were assumed dead and dissected. Pupae from which parasitoids had emerged were then left at room temperature for 48 hr to ensure complete wasp eclosion.

### 2.5 Statistical analysis

All response variables were tested using generalized linear mixed models. We included the family and the larval container nested within each family as random factors to test objectives (i) and (ii). For objective (iii), larval family and parasitoid location of origin, but not larval container, were included as random factors (due to data limitation).

The effect of rearing density (objective i) on the variable assessing survival to pupation was analyzed with binomial error distribution and logit link function, and density as fixed effect. Larval development time (the number of days from breaking diapause until pupation) and pupal weight were assessed with density treatment (low and high), sex and their interaction as fixed effects. As sex can only be determined from adults, all models including sex are performed on a set of data including only individuals that reached adulthood. In the analysis of pupal weight, we additionally included larval development time as covariate. For pupal development time (the number of days from pupation until adult eclosion), we included density treatment, sex, pupal treatment (naïve, encapsulation only, parasitism only, and encapsulation followed by parasitism), all second‐order interactions as fixed factors, and pupal weight as covariate.

Pupal encapsulation (objective ii) was analyzed using a similar model plus larval development time as covariate. Adult encapsulation was analyzed with a model including density, sex, pupal treatment (*N*,* E*,* P,* and *EP*), and all second‐order interactions. A separate model with adult encapsulation as response variable and pupal encapsulation as fixed effect was run to test for correlation between the two assays (*E* and *EP* groups only; see Table S1). Adult survival, defined as successful eclosion from the pupal stage, was analyzed using a binomial error distribution and logit link function. Density, pupal treatment, and their interaction were included as fixed effects, and pupal weight as covariate. In order to test for the effects of pupal encapsulation on adult survival, we ran a separate model on a smaller set of data where pupal encapsulation was the only fixed effect.

All the wasp offspring‐related traits (objective iii) were assessed on a subset of data including only the parasitized pupae (groups *P* and *EP*) that did not survive parasitism. To model wasp brood size (number of alive plus dead wasps), we included density treatment, pupal treatment, second‐order interactions among these, and pupal weight as covariate. A similar model was used for the analyses of proportion of wasps eclosing, and wasp brood sex ratio. As they were both concatenated variables represented by the number of wasps alive over the dead, and the number of males over the females, respectively, they were modeled with a binomial error distribution. As performed above, a separate model on a smaller set of data (only *EP* treatment) including only pupal encapsulation was performed to detect effects of previous investment in immunity on wasp eclosion or sex ratio.

To assess the amount of variation explained by larval family (objective iv) in contrast to common environment (larval container), or parasitoid genetic background (wasp origin), we calculated intraclass correlation coefficients for each of the random factors used in the models presented. This information is shown for the models that best explain the response variables (Table 2).

Model selection was performed by comparing the full model and alternative models based on the AICs (ML fit). *p*‐Values were obtained from ANOVAs on the model with the lowest AIC (REML refit; Table S1). In some cases, the best model differed less than two AIC points from others. *p*‐Values for these alternative models are in Table S1. All data were analyzed with R (v 3.3.1) Mixed Models packages “lme4” and “lmerTest” (Bates, Mächler, Bolker, & Walker, 2015).

## 3 RESULTS

### 3.1 Density and performance of *Melitaea cinxia* (i)

**Figure 3:**
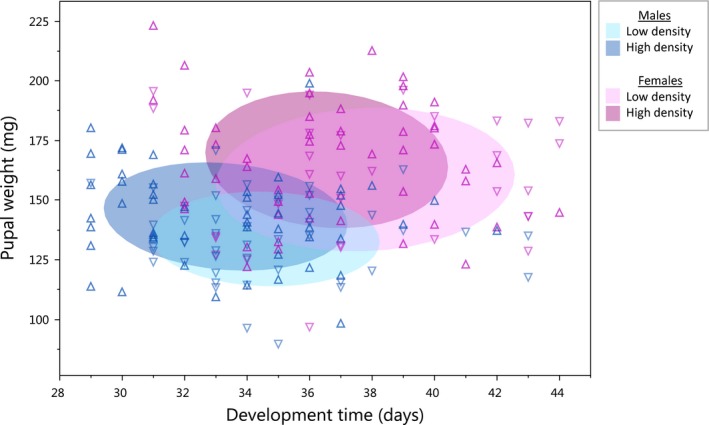
Pupal weight by larval development time (from exiting the diapause to pupation) in relation to sex (*p *<* *.005 for both) and density treatment (*p *<* *.05 for both). Males and females are represented by blue and red, respectively, and high and low density by dark and light shading (coverage of 50% of the observations), respectively. Up‐ and downward facing triangles represent individuals reared in high and low density, respectively

Males had a shorter larval development time (*F*_1,171.5_ = 41.5, *p *<* *.0001; Figure 3; Table S1) and weighed less as pupae than females (*F*_1,171.3_ = 70.8, *p *<* *.0001; Figure 3). Larvae that were reared at high density completed their development on average 1 day earlier than those reared at low density (*F*_1,51.5_ = 5.3, *p *=* *.03; Figure 3), and were heavier as pupae (*F*_1,70.9_ = 4.6, *p *=* *.04; Figure 3; Table 1). In addition, larvae that developed quickly were generally heavier as pupae (*F*_1,174.7_ = 5.3, *p *=* *.02; Table S1). Heavier pupae reached the adult stage more slowly than lighter ones (*F*_1,173_ = 7.3, *p *=* *.007; Table S1). Rearing density did not affect larval survival to the pupal stage (*p *>* *.8).

**Table 1:** Mean (±*SE*), and percentage values of *M. cinxia* and *P. apum* life‐history traits following the larval high‐density (HD) and low‐density (LD) treatments, and the pupal naïve (*N*), encapsulation (*E*), parasitism (*P*), and encapsulation and parasitism (*EP*) treatments

	Larval treatment	Pupal treatment			
		*N*	*E*	*P*	*EP*
Sample size (after pupation)	LD	41	39	37	37
	HD	44	48	50	47
Development time larva–pupa (days)	LD	36.5 (0.6)	36.4 (0.6)	36.0 (0.7)	36.1 (0.6)
	HD	35.0 (0.5)	35.3 (0.5)	35.5 (0.5)	35.5 (0.5)
Pupal weight (mg)	LD	144.5 (3.3)	147.9 (5.1)	147.0 (4.2)	147.6 (3.9)
	HD	151.5 (4.4)	154.5 (3.9)	148.2 (4.3)	157.3 (3.8)
Pupal encapsulation rate (black percentage per pixel)	LD	–	66.3 (2.6)	–	66.2 (2.3)
	HD	–	69.6 (2.0)	–	67.6 (2.0)
Development time pupa–adult (days)	LD	7.5 (0.1)	7.6 (0.1)	7.8 (0.3)	7.9 (0.2)
	HD	7.8 (0.1)	7.7 (0.1)	7.4 (0.2)	7.8 (0.1)
Butterflies eclosed (% of pupae)	LD	95.1	84.6	10.8	27.8
	HD	93.3	79.2	28	27.7
Adult encapsulation rate (black percentage per pixel)	LD	57.1 (2.1)	55.7 (2.7)	57.9 (5.2)	58.9 (3.4)
	HD	57.0 (2.3)	56.6 (2.2)	64.6 (3.2)	57.9 (3.0)
Average wasp brood size	LD	–	–	19.4 (1.4)	18.5 (2.0)
	HD	–	–	19.7 (1.7)	19.9 (2.0)
Eclosed wasps (% of brood)	LD	–	–	82.3	67.7
	HD	–	–	57.7	53
Wasp sex ratio (male % of brood)	LD	–	–	70.9	40
	HD	–	–	50	47.5

### 3.2 Density, butterfly immunity, and pupal survival (ii)

**Figure 4:**
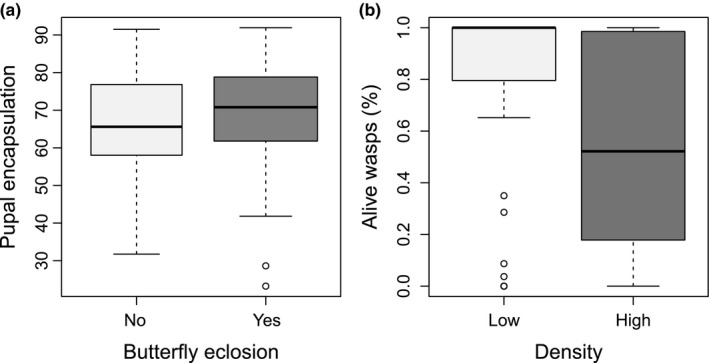
Pupal encapsulation rate of individuals that did or did not survive until adulthood (a) and percentage of wasps per brood eclosed from pupae reared in low or high density (*p *=* *.02; b). Butterflies that did and did not survive (a), and high and low density (b) are each represented by dark and light gray, respectively

All pupae and adults treated with the nylon filaments exhibited some encapsulation. Rearing density, sex, and pupal weight did not influence pupal encapsulation rate (*p *>* *.3 for all). However, larvae that developed quickly had high pupal encapsulation rates (*F*_1,75.5_ = 11.8, *p *=* *.001; Table S1). The pupal treatment (naïve, encapsulation assay, parasitized, or parasitized and encapsulated) influenced the fraction of pupae surviving to adulthood, with the highest survival in the naïve group, and the lowest among those exposed to parasitism (~94% and ~23%, respectively; Tukey post hoc, *p *<* *.05 for all; Table 1). Experiencing the encapsulation assay before being parasitized (i.e., priming) did not affect surviving parasitism (Tukey post hoc between *P* and *EP*,* p *>* *.6). Although density had no effect on survival to adulthood, a Fisher's test within each pupal treatment indicated marginally lower survival to adulthood by unprimed individuals that encountered the parasitoid, but only at the low rearing density (*p *=* *.06; *p *>* *.5 for the rest; Table 1). There was also a suggestive trend toward high survival to adulthood by pupae with high encapsulation rates (*df* = 1, Χ = 3.6, *p *=* *.06; Figure 4a; Table S1).

**Figure 5:**
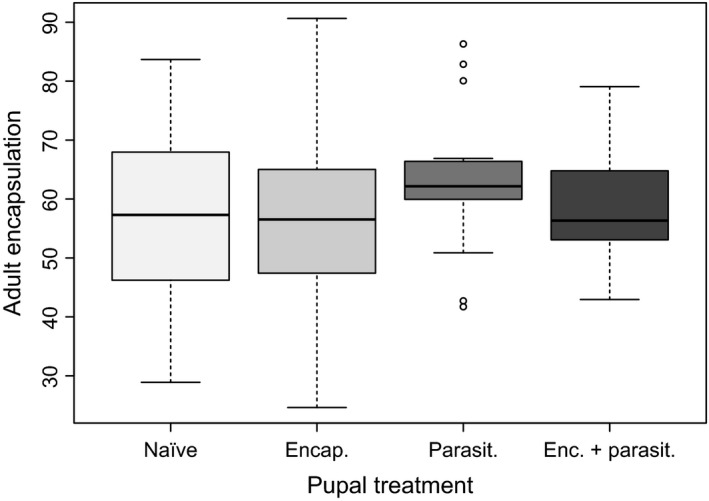
Adult encapsulation by pupal treatment. More melanized filaments are represented by larger numbers. Encapsulation rate in the parasitoid only group is significantly higher than the naïve and encapsulation only groups (*p *<* *.01), but not than the group including both encapsulation and parasitism (*p *>* *.6)

Adult encapsulation was significantly higher in unprimed individuals that had survived parasitism compared to unparasitized individuals (Tukey post hoc, *N*‐*P*,* p *=* *.05; *E*‐*P*,* p *=* *.01; Figure 5). However, adult encapsulation did not differ between primed and unprimed parasitized individuals (Tukey post hoc, *p *>* *.4; Figure 5), and it was unaffected by rearing density or sex (*p *>* *.1 for both). Finally, adult encapsulation was positively correlated with pupal encapsulation (*F*_1,75.9_ = 10, *p *=* *.002; Fig. S1).

### 3.3 Density and parasitoid performance (iii)

Parasitoids successfully developed in almost 62% of the parasitized pupae, with an average surviving brood size of about 19 individuals. On average, 65% of the individuals in a brood successfully emerged from the parasitized pupae. Wasp brood size increased with host pupa size (pupal weight, *F*_1,100.4_ = 5.6, *p *=* *.02), with no detectable effect of rearing density, pupal treatment, or pupal encapsulation (*p *>* *.2 for all). The ratio of alive to dead wasps in a brood was lower in the hosts reared at high density compared to the low‐density ones, with no detectable difference between pupal treatments (density, *df* = 1, Χ = 5.7, *p *=* *.02, Figure 4b; pupal treatment, *p *>* *.5; Table S1). Additionally, a higher ratio of alive wasps eclosed from small host pupae than from larger ones (*df* = 1, Χ = 5.4, *p *=* *.02; Table S1). The ratio of alive wasps was unaffected by host pupal encapsulation response (*p *>* *.5).

The sex ratio of the emerging parasitoid broods was male biased (70%) only in the unprimed hosts that had been reared at low density (pupal treat × density, *df* = 1, Χ = 4.3, *p *=* *.04; Table S1). In the remaining categories, the sex ratio of eclosing wasps was between 40% and 50% male (Table 1).

### 3.4 Butterfly family versus common environment or wasp origin effects (iv)

For butterfly‐related traits, the proportion of variance explained by the postdiapause common environment (i.e., container nested within larval family) was on average ~6.5% and ranged between 0% for pupal development time and pupal encapsulation, and 23.8% for adult encapsulation rate (Table 2). The proportion of variance explained by the larval family was on average ~13%. It ranged from 2.4% for adult survival to 23% for larval development time (Table 2). In most of the cases, the family accounted for a greater proportion of the variance than did the postdiapause common environment. Notably, however, adult survival and adult encapsulation rate showed a reverse trend, with greater variation among containers within the same family than among families (Table 2).

**Table 2:** Intraclass correlation coefficients (ICC) calculated for the random effects of the best model explaining the response variable

Response variable	Random factors	ICCs (%)
Larval development time	Larval container [butterfly family]	3.4
	Butterfly family	23
Survival to pupation	Larval container [butterfly family]	11.2
	Butterfly family	12.3
Pupal weight	Larval container [butterfly family]	2.6
	Butterfly family	19.9
Pupal development time	Larval container [butterfly family]	0
	Butterfly family	3.2
Pupal encapsulation	Larval container [butterfly family]	0
	Butterfly family	14.1
Adult survival	Larval container [butterfly family]	4.8
	Butterfly family	2.4
Adult encapsulation	Larval container [butterfly family]	23.8
	Butterfly family	16.4
Wasp brood size (alive + dead)	Wasp location of origin	6.4
	Butterfly family	0
Live to dead brood ratio	Wasp location of origin	0
	Butterfly family	11.6
Wasp brood sex ratio	Wasp location of origin	17.6
	Butterfly family	0

For wasp brood‐related traits, the proportion of variance explained by the location of origin of the foundress wasps was ~6% for brood size, and almost 18% for wasp sex ratio (Table 2). No variance was explained by the host larval family, except for the ratio of viable wasps that eclosed from the pupae, which was almost 12%. In this case, the wasp origin explained none of the variance (Table 2).

## 4 DISCUSSION

Our interest was to determine the effects of crowding on investment in life‐history traits and immunity in a presocial insect. We expected a trade‐off between immunity and life‐history traits if the interaction among nest mates was stressful (Figure 1, hyp. I). Conversely, if crowding induced beneficial, cooperative, interactions among nest mates, we expected no trade‐offs (Figure 1, hyp. II). Our findings generally supported the second hypothesis and hence a general benefit of high density in the Glanville fritillary butterfly.

We found that larvae reared at high density grew faster and reached larger pupal mass, with no impact on survival to the pupal stage. Hence, our results indicate that individuals benefit from crowding, at least when they are not food limited. This could be the result of an increase in growth due to increased density inducing individuals to feed at higher rate. Such behavioral response may be adaptive under natural conditions, where high density is generally associated with limited food availability (Sillanpää, 2008). Alternatively, individuals could actually benefit from cooperative interactions among group members if, for example, a higher number of larvae promotes piercing the leaf cuticle faster or facilitates inactivation of plant defensive compounds, or promotes thermoregulation (Bryant, Thomas, & Bale, 2000; Fitzgerald & Costa, 1999; Lawrence, 1990; Tsubaki, 1981).

There was no strong impact of crowding on the ability of individuals to survive parasitism. However, individuals reared at low density had marginally lower survival when they were not primed (low density‐parasitized group [LD‐P], Table 1). Additionally, the group that experienced only parasitism showed the highest adult encapsulation rate (significant difference in comparison with groups that were not parasitized; Figure 5). The latter suggests that being parasitized triggers host immunity to an extent that is still evident at the adult stage. A similar pattern was found in dragonfly larvae in response to predation (Duong & McCauley, 2016). The difference in surviving parasitism between unprimed individuals from low and high density may result from the immune upregulation in response to parasitism imposing a physiological cost on the apparently weaker low density‐parasitized pupae (LD‐P group) (Moret & Schmid‐Hempel, 2000; reviewed in Zuk & Stoehr, 2002). Melanin production in the process of encapsulation is known to increase oxidative stress, which can be life‐threatening for weak individuals (Sadd & Siva‐Jothy, 2006).

Furthermore, even though crowding did not affect the total brood size of the parasitoid, it did influence the sex ratio of the wasps as well as the ratio of successfully emerging adult wasps. Strongly male biased parasitoid broods came from the low‐density group (unprimed only; Table 1), while broods from the other groups had on average a 1:1 sex ratio, suggesting that the unprimed low‐density individuals were perceived as low‐quality hosts by the ovipositing parasitoids. Parasitoid wasps can control the sex of the offspring during oviposition (Frank, 1983; Slansky, 1986). In general, male parasitoids are smaller than females and thus require fewer resources for development, and a male sex bias of broods laid by parasitoids is often thought to reflect poor host quality (West, 2009). In a similar vein, the difference in the ratio of successfully emerging wasps between the density treatments indicates higher wasp mortality in the high‐density group. Thus, despite butterfly survival not being affected by the density treatment, these results suggests that the pupae from high‐density treatment showed a greater “immune attempt” to resist the parasitoids, possibly as a consequence of their fitter condition (Laurentz et al., 2012).

Contrary to our expectation of immune priming being beneficial, in adult butterflies encapsulation of the primed group (*EP*) was not upregulated. Instead, it was lower than in the unprimed group. This is not due to deleterious effects of the encapsulation assay, or physiological costs of repeated immune activation, as host survival rate in the primed group was ~8% higher than in the unprimed group (*EP* and *P* groups, Table 1). The result indicates that immune priming may be irrelevant or involve other components of the insect immune system than the encapsulation response. Immune priming also had no impact on the parasitism success by *P. apum* in terms of total wasp brood size, or number of live wasps eclosing from the host.

Finally, consistent with previous work on the Glanville fritillary butterfly (development; Laine, 2004; and immunity; van Nouhuys, Niemikapee, & Hanski, 2012; Saastamoinen, Hirai, & van Nouhuys, 2013), we show that family rather than the common postdiapause environment explains a relatively high proportion of the total variance in most of the traits measured. The family effect assessed includes the genetic background as well as the common prediapausing and diapausing environment, and any parental effects. Only adult survival and adult encapsulation rate were influenced more by the common postdiapause environment than by family background. The influence of common postdiapause environment on adult immunity may be explained by the production of antimicrobial compounds in larval exudates, or in the silk, as found in other presocial and eusocial insects (Arce, Smiseth, & Rozen, 2013; Tranter & Hughes, 2015).

From the parasitoid side, butterfly family played no role on wasp brood size and sex ratio, which were instead shaped by wasp population of origin. This result is not surprising, given the ability of parasitoid wasps to adjust brood size and sex ratio (Frank, 1983; Slansky, 1986). In contrast, the proportion of wasps eclosed was primarily determined by butterfly family, highlighting the importance of host genetic background in the outcome of host–parasite interactions, as was shown for another parasitoid of *M. cinxia* (van Nouhuys et al., 2012).

## 5 CONCLUSIONS

We found that *M. cinxia* larvae developed faster and reached a larger pupal mass when reared at high density, suggesting a positive effect of crowding. Moreover, high‐density individuals showed a greater immune attempt by killing a larger fraction of parasitoids per brood than did those reared in low density. Although a butterfly can only survive if it kills the entire parasitoid brood, this result indicates that the ability to fight a parasite is one of the benefits of group living, in the absence of a trade‐off with other life‐history traits. Finally, we found a role of the common postdiapause environment experienced by developing larvae in structuring immunity and survival to adulthood. Our study demonstrates that this presocial insect has evolved to perform better under more crowded conditions (i.e., hyp. II in Figure 1) as, at least under ad libitum food conditions, it does not show indications of stress often observed in solitary insects.

## CONFLICT OF INTEREST

None declared.

## DATA ACCESSIBILITY

The data supporting the research have been archived in the “Dryad” public repository under the provisional data identifier: https://doi.org/10.5061/dryad.3c457.

## AUTHORS’ CONTRIBUTIONS

ER, MS, and SvN conceived the ideas and designed methodology; ER collected and analyzed the data; ER, MS, and SvN wrote the manuscript. All authors contributed critically to the drafts and gave final approval for publication.
