# Supplementary material for: The more the merrier: Conspecific density improves performance of gregarious larvae and reduces susceptibility to a pupal parasitoid
Source: Ecol Evol. 2017 Nov 7;7(24):10710–20. doi: 10.1002/ece3.3571 (PMC5743493; doi:10.1002/ece3.3571)

**Supplementary material Appendix 1**

**Table S1.** Model selection based on AIC, significance values for the best model and for alternative models to the one with the lowest AIC (when Δ AIC < 2). Effect sizes between low and high density treatments are presented for each response variable as Cohen’s d.

| **Response variable** | | **Model selection** | | | | | | | **AIC** | | | | | | | | | | | | | |  | | | | |
| --- | --- | --- | --- | --- | --- | --- | --- | --- | --- | --- | --- | --- | --- | --- | --- | --- | --- | --- | --- | --- | --- | --- | --- | --- | --- | --- | --- |
| Larval development time  Cohen’s d = -0.27 (small) | | sex  density  density + sex  density + sex + density*sex | | | | | | | 929.2  961.7  926  927.8 | | | | | | | | | | | | | |  | | | | |
|  | | | | | | | | | | | | | | | | | | | | | | | | | | | |
|  | | **Final model:** | | | | | | | **Df** | | | **Df den** | | | **F value** | | | | | | | **Pr(>F)** |  | | | | |
|  | |  | | density  sex | | | | | 1  1 | | | 51.5 171.5 | | | 5.3  41.5 | | | | | | | 0.03  <0.0001 | *  *** | | | | |
|  | | | | | | | | | | | | | | | | | | | | | | | | | | | |
| **Response variable** | | **Model selection** | | | | | | **AIC** | | | | | | | | | | | | | | |  | | | | |
| Survival to pupation  Cohen’s d = 0.006 (negligible) | | density | | | | | | 1826.1 | | | | | | | | | | | | | | |  | | | | |
|  | | | | | | | | | | | | | | | | | | | | | | | | | | | |
|  | | **Final model:** | | | | | | | **Df** | | | **Chi df** | | | **Chisq** | | | | | | | **Pr(>Chisq)** | | | |  | |
|  | |  | | density | | | | | 1 | | | 1 | | | 0.0566 | | | | | | | 0.812 |  | | | | |
|  | | | | | | | | | | | | | | | | | | | | | | | | | | | |
| **Response variable** | | **Model selection** | | | | | | | | | | **AIC** | | | | | | | | | | |  | | | | |
| Pupal weight  Cohen’s d = 0.43 (small) | | sex  density  larva dev time  density + sex  density + sex + density*sex  larva dev time + density + sex  larva dev time + density + sex + density*sex | | | | | | | | | | 1609.0  1658.6  1664.3  1604.6  1606.5  1601.8  1603.7 | | | | | | | | | | |  | | | | |
|  | | | | | | | | | | | | | | | | | | | | | | | | | | | |
|  | | **Final model:** | | | | | | | **Df** | | | **Df den** | | | **F value** | | | | | | | **Pr(>F)** |  | | | | |
|  | |  | | larva dev time  density  sex | | | | | 1  1  1 | | | 174.7  70.9  171.3 | | | 5.3  4.6  70.8 | | | | | | | 0.02  0.04  <0.0001 | *  *  *** | | | | |
|  | | **Alternative model:** | | | | | | | **Df** | | | **Df den** | | | **F value** | | | | | | | **Pr(>F)** |  | | | | |
|  | |  | | larva dev time  density  sex  density*sex | | | | | 1  1  1  1 | | | 173.7  108.5  163  166.2 | | | 5.2  3.3  9.7  0.1 | | | | | | | 0.02  0.07  0.002  0.7 | *  .  ** | | | | |
|  | | | | | | | | | | | | | | | | | | | | | | | | | | | |
| **Response variable** | | **Model selection** | | | | | | | | | | | | | **AIC** | | | | | | | |  | | | | |
| Pupal development time  Cohen’s d = 0.29 (small) | | density  sex  pupal weight  density + sex + density*sex  pupal treatment  density + pupal treat + sex + pupal weight  density + pupal treat + density*pupal treat  pupal treat + sex + pupal treat*sex  density + pupal treat + sex + pupal weight +  density*sex + density*pupal treat + pupal treat*sex | | | | | | | | | | | | | 342.4  345.2  338.5  344.9  346.4  343.4  347.2  345  345.4 | | | | | | | |  | | | | |
|  | | | | | | | | | | | | | | | | | | | | | | |  | | | | |
|  | | **Final model:** | | | | | | | **Df** | | | **Df den** | | | **F value** | | | | | | | **Pr(>F)** |  | | | | |
|  | |  | | pupal weight | | | | | 1 | | | 173 | | | 7.3 | | | | | | | 0.007 | ** | | | | |
| **Response variable** | | **Model selection** | | | | | | | | | | | | | | | | **AIC** | | | | |  | | | | |
| Pupal encapsulation  Cohen’s d = 0.05 (negligible) | | sex  density  pupal weight  larva dev time  density + sex  density + sex + density*sex  pupal weight + larva dev time + density + sex  pupal weight + larva dev time + density + sex + density*sex | | | | | | | | | | | | | | | | 655.4  656.3  656.2  645.0  657.3  658.5  649.6  649.4 | | | | |  | | | | |
|  | | | | | | | | | | | | | | | | | | | | | | | | | | | |
|  | | **Final model:** | | | | | | | **Df** | | | **Df den** | | | **F value** | | | | | | | **Pr(>F)** |  | | | | |
|  | |  | | larva dev time | | | | | 1 | | | 75.7 | | | 11.8 | | | | | | | 0.001 | ** | | | | |
|  | | | | | | | | | | | | | | | | | | | | | | | | | | | |
| **Response variable** | | **Model selection** | | | | | | | | | | | | | | | **AIC** | | | | | |  | | | | |
| Adult survival  Cohen’s d = -0.005 (negligible) | | density  pupal weight  pupal treat  density + pupal treat + pupal weight  density + pupal treat + density*pupal treat  density + pupal treat + pupal weight + density*pupal treat | | | | | | | | | | | | | | | 476.4  475.7  313  315.7  315.2  316.3 | | | | | |  | | | | |
|  | | | | | | | | | | | | | | | | | | | | | | | | | | | |
|  | | **Final model:** | | | | | | | **Df** | | | **Chi df** | | | **Chisq** | | | | | | | **Pr(>Chisq)** | | |  | | |
|  | |  | | pupal treat | | | | | 3 | | | 3 | | | 167.38 | | | | | | | <0.0001 | | *** | | | |
|  | | | | | | | | | | | | | | | | | | | | | | | | | | | |
| **Response variable** | **Model selection** | | | | | **AIC** | | | | | | | | | | | | | | | | |  | | | | |
| Adult survival  (groups *E*, *EP* only) | pupal encapsulation | | | | | 233.3 | | | | | | | | | | | | | | | | |  | | | | |
|  | **Final model:** | | | | | | | **Df** | | | | **Chi df** | | | **Chisq** | | | | | | | **Pr(>Chisq)** | | |  | | |
|  |  | | | | pupal encapsulation | | | | | 1 | | | 1 | 3.5864 | | | | | | | 0.05826 | | | | . | | |
|  | | | | | | | | | | | | | | | | | | | | | | | | | | | |
| **Response variable** | | **Model selection** | | | | | | | | | | | | | | | | | | **AIC** | | | | | | |  |
| Adult encapsulation  Cohen’s d = 0.13 (negligible) | | sex  density  sex+ density + density*sex  pupal treatment  pupal treat + density + pupal treat*density  pupal treat + sex + pupal treat*sex  pupal treat + density + sex  pupal treat + density + sex + pupal treat*density + pupal treat*sex + density*sex | | | | | | | | | | | | | | | | | | 1412.4  1413.5  1415.2  1408.5  1414.2  1410.3  1410.3  1418.6 | | | | | | |  |
|  | | | | | | | | | | | | | | | | | | | | | | | | | | | |
|  | | **Final model:** | | | | | | | **Df** | | **Df den** | | | | **F value** | | | | | | | **Pr(>F)** |  | | | | |
|  | |  | | pupal treat | | | | | 3 | | 129.7 | | | | 3.5 | | | | | | | 0.02 | * | | | | |
|  | | **Alternative models:** | | | | | | | **Df** | | **Df den** | | | | **F value** | | | | | | | **Pr(>F)** |  | | | | |
|  | |  | | pupal treat  sex  pupal treat*sex  pupal treat  density  sex | | | | | 3  1  3  **Df**  3  1  1 | | 126.7  139.9  141.4  **Df den**  127.5  44.3  153.5 | | | | 3.8  2.1  1.4  **F value**  3.3  0.3  1.8 | | | | | | | 0.01  0.1  0.2  **Pr(>F)**  0.02  0.6  0.2 | *  * | | | | |
|  | | | | | | | | | | | | | | | | | | | | | | | | | | | |
| **Response variable** | **Model selection** | | | | | | | | | | | **AIC** | | | | | | | | | | |  | | | | |
| Adult encapsulation  (groups *E*, *EP* only) | pupal encapsulation | | | | | | | | | | | 652.5 | | | | | | | | | | |  | | | | |
|  | **Final model:** | | | | | | | **Df** | | | **Df den** | | | | **F value** | | | | | | | **Pr(>F)** |  | | | | |
|  |  | | pupal encapsulation | | | | | 1 | | | 75.9 | | | | 10 | | | | | | | 0.002 | ** | | | | |
| **Response variable** | | **Model selection** | | | | | | | | | | | | | | | | | **AIC** | | | |  | | | | |
| Wasp brood size  (alive + daead wasps, groups *P*, *EP*)  Cohen’s d = 0.09 (negligible) | | pupal treatment  density  pupal weight  density + pupal treat + density*pupal treat  density + pupal treat + pupal weight  density + pupal treat + pupal weight + density*pupal treat | | | | | | | | | | | | | | | | | 747.5  747.4  741.8  751.2  745.6  747.6 | | | |  | | | | |
|  | | | | | | | | | | | | | | | | | | | | | | | | | | | |
|  | | **Final model:** | | | | | | | **Df** | | | **Df den** | | | **F value** | | | | | | | **Pr(>F)** |  | | | | |
|  | |  | | pupal weight | | | | | 1 | | | 100.4 | | | 5.6 | | | | | | | 0.02 | * | | | | |
|  | | | | | | | | | | | | | | | | | | | | | | | | | | | |
| **Response variable** | | **Model selection** | | | | | **AIC** | | | | | | | | | | | | | | | |  | | | | |
| Wasp brood size  (alive + dead wasps, group *EP* only) | | pupal encapsulation | | | | | 337.7 | | | | | | | | | | | | | | | |  | | | | |
|  | | | | | | | | | | | | | | | | | | | | | | | | | | | |
|  | | **Final model:** | | | | | | | **Df** | | | **Df den** | | | **F value** | | | | | | | **Pr(>F)** |  | | | | |
|  | |  | | pupal encapsulation | | | | | 1 | | | 39.8 | | | 1.7 | | | | | | | 0.2 |  | | | | |
|  | | | | | | | | | | | | | | | | | | | | | | | | | | | |
| **Response variable** | | **Model selection** | | | | | | | | | | | | | | | | | **AIC** | | | |  | | | | |
| Alive wasps ratio  (groups *P*, *EP*)  Cohen’s d = -0.54 (medium) | | pupal treatment  density  pupal weight  density + pupal treat + pupal weight  density + pupal treat + density*pupal treat  density + pupal treat + pupal weight + density*pupal treat | | | | | | | | | | | | | | | | | 470.5  462.6  463  460.7  466  462.7 | | | |  | | | | |
|  | | | | | | | | | | | | | | | | | | | | | | | | | | | |
|  | | **Final model:** | | | | | | | **Df** | | | **Chi df** | | | **Chisq** | | | | | | | **Pr(>Chisq)** | | | |  | |
|  | |  | | pupal weight  density  pupal treat | | | | | 1  1  1 | | | 1  1  1 | | | 5.37  5.69  0.3 | | | | | | | 0.02  0.017  0.58 | | | | *  * | |
|  | | **Alternative models:** | | | | | | | **Df** | | | **Chi df** | | | **Chisq** | | | | | | | **Pr(>Chisq)** | | | |  | |
|  | |  | | density  density  pupal treat  pupal weight  density*pupal treat | | | | | 1  **Df**  1  1  1  1 | | | 1  **Chi df**  1  1  1  1 | | | 9.3  **Chisq**  0.2  0.06  5.4  0.006 | | | | | | | 0.002  **Pr(>Chisq)**  0.7  0.8  0.02  0.9 | | | | **  * | |
|  | |  | |  | | | | |  | | |  | | |  | | | | | | |  | | | |  | |
| **Response variable** | | **Model selection** | | | | | | | | | | | | | | **AIC** | | | | | | |  | | | | |
| Alive wasps ratio  (group *EP* only) | | pupal encapsulation | | | | | | | | | | | | | | 215.3 | | | | | | |  | | | | |
|  | | | | | | | | | | | | | | | | | | | | | | | | | | | |
|  | | **Final model:** | | | | | | | **Df** | | | **Chi df** | | | **Chisq** | | | | | | | **Pr(>Chisq)** | | |  | | |
|  | |  | | pupal encapsulation | | | | | 1 | | | 1 | | | 0.3 | | | | | | | 0.59 | | |  | | |
|  | | | | | | | | | | | | | | | | | | | | | | | | | | | |
| **Response variable** | | **Model selection** | | | | | | | | | | | | | | | | | **AIC** | | | |  | | | | |
| Wasp sex-ratio  Cohen’s d = -0.4 (small) | | pupal treatment  density  pupal weight  density + pupal treat + pupal weight  density + pupal treat + density*pupal treat  density + pupal treat + pupal weight + density*pupal treat | | | | | | | | | | | | | | | | | 383.6  385.2  388.2  383.1  380.3  381.2 | | | |  | | | | |
|  | | | | | | | | | | | | | | | | | | | | | | | | | | | |
|  | | **Final model:** | | | | | | | **Df** | | | **Chi df** | | | **Chisq** | | | | | | | **Pr(>Chisq)** | | |  | | |
|  | |  | | pupal treat  density  density*pupal treat | | | | | 1  1  1 | | | 1  1  1 | | | 6.9  5.3  4.3 | | | | | | | 0.0087  0.021  0.037 | | | **  *  * | | |
|  | | **Alternative model:** | | | | | | | **Df** | | | **Chi df** | | | **Chisq** | | | | | | | **Pr(>Chisq)** | | |  | | |
|  | |  | | pupal treat  density  pupal weight  density*pupal treat | | | | | 1  1  1  1 | | | 1  1  1  1 | | | 6.7  5.1  1.1  3.96 | | | | | | | 0.01  0.024  0.3  0.046 | | | *  *  * | | |
|  | | | | | | | | | | | | | | | | | | | | | | | | | | | |

**Figure S1.** Positive correlation between pupal and adult encapsulation rates (*P*=0.002).


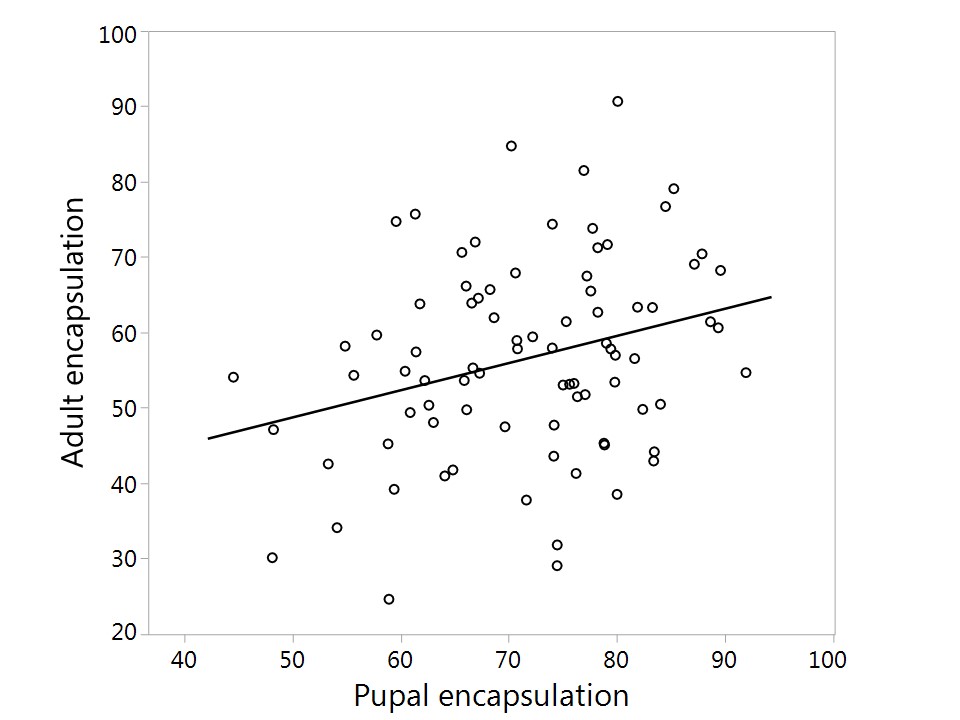

Supplement: Supplementary file 1 [file ECE3-7-10710-s001.docx]
